# Variations in association of nasal microbiota with virulent and non-virulent strains of *Glaesserella* (*Haemophilus*) *parasuis* in weaning piglets

**DOI:** 10.1186/s13567-020-0738-8

**Published:** 2020-02-03

**Authors:** Yasser S. Mahmmod, Florencia Correa-Fiz, Virginia Aragon

**Affiliations:** 1 IRTA, Centre de Recerca en Sanitat Animal (CReSA, IRTA-UAB), Campus de la Universitat Autònoma de Barcelona, 08193 Bellaterra, Spain; 2 Universitat Autònoma de Barcelona, Cerdanyola del Vallès, 08193 Bellaterra, Barcelona Spain; 3 Infectious Diseases, Department of Animal Medicine, Faculty of Veterinary Medicine, Zagazig University, Zagazig, Sharkia 44511 Egypt; 4 OIE Collaborating Centre for the Research and Control of Emerging and Re-emerging Swine Diseases in Europe (IRTA-CReSA), Bellaterra, Barcelona Spain

## Abstract

*Glaesserella* (formerly *Haemophilus*) *parasuis* causes Glässer’s disease, which results in high economic loss in the swine industry. To understand the polymicrobial interactions of *G. parasuis* and the nasal microbiota, the statistical association patterns of nasal colonizing bacteria with virulent and non-virulent strains of *G. parasuis* were studied accounting for the farm management practices as potential risk factors for the occurrence of Glässer’s disease. The nasal microbiota from 51 weaned-piglets from four farms with Glässer’s disease and three farms with no respiratory diseases was previously characterized and included in this study. The presence of virulent and/or non-virulent *G. parasuis* strains in the nasal cavities was determined in order to establish the potential association with other members of the nasal microbiota. Multivariate logistic and linear regression models were performed among the various members of nasal microbiota and *G. parasuis*. The multi-site production system and disease presence in the farm were both significantly associated with the presence of *G. parasuis* virulent strains in the nose of the piglets. Differential bacterial associations were observed with virulent or non-virulent *G. parasuis*. *Chitinophagaceae*, *Corynebacteriaceae* and *Corynebacterium* were positively associated with the virulent *G. parasuis* strains, while *Enterobacteriaceae, Peptostreptococcaceae*, *Clostridium XI,* and *Escherichia/Shigella* were negatively associated with virulent *G. parasuis*. On the other hand, *Flavobacteriaceae*, *Planobacterium*, and *Phascolarctobacterium* were positively associated with the non-virulent *G. parasuis* strains, while *Rikenellaceae*, *Enterococcaceae*, *Odoribacter*, and *Corynebacterium* were negatively associated with non-virulent *G. parasuis*. In conclusion, the nasal microbiota communities showed variations in the association with the *G. parasuis* strains type.

## Introduction

The nasal mucosa of piglets is colonized by a wide array of bacteria, the nasal microbiota, many of which are still unknown [1]. The microbiota establishes mutual relationship with its host and the resulting cross-talk extends beyond the balance between tolerance to commensal microorganisms and developing protection against pathogens [2]. *Glaesserella parasuis* (formerly *Haemophilus parasuis* [3]) can be found among the microbes widely distributed in swine population, colonizing the upper respiratory tract of healthy piglets, particularly the nasal cavity [4, 5]. *G. parasuis* causes Glässer’s disease, which results in one of the highest economic losses in nursery piglets, but the factors influencing its development are not totally understood [1, 6]. Glässer’s disease is commonly developed after weaning due to a reduction in maternal antibodies, mix of litters, strains variations, and other stress factors [7, 8]. Basically, all pig farms are positive to *G. parasuis*, but not all farms develop the Glasser’s disease symptoms.

Recently, through metagenomics techniques it was revealed how “an equilibrated” microbiota affects potential pathogens [9, 10]. Metagenomics techniques could reveal the host-microbe and microbe–microbe interactions toward a predictive, dynamic ecosystem model. Recent studies on gut microbiota have provided the evidence that the onset of a disease can be the result of a change in the interaction with other microorganisms [11]. Therefore, understanding the polymicrobial interactions of pathogens, such as *G. parasuis*, and the microbiota in the nasal cavity is crucial for the following reasons (a) colonization is the initial step of the disease process, (b) colonized piglets serve as reservoirs for bacterial transmission to others in the population and, (c) microbiome structure may have an effect on development of Glässer’s disease.

Several recent studies uncovered the nasal microbiota composition in pigs and their association with different infectious pathogens [1, 12–14]. Strube et al. [14] showed that the porcine nasal mucosa harbored *Rothia* and had higher relative levels of *Streptococcus* and *Moraxella*, while levels of *Aerococcus*, *Facklamia* and *Jeotgalibaca* were relatively lower. Espinosa-Gongora et al. [13] reported that the nasal microbiota may play a role in the individual predisposition to *Staphylococcus aureus* nasal carriage in pigs. On the contrary, Weese et al. [12] found no impact of the nasal microbiota on the methicillin-resistant *Staphylococcus aureus* carriage in pigs. *G. parasuis* is a heterogeneous bacterial pathogen encompassing both virulent and non-virulent strains. Keeping that in mind, virulent strains of *G. parasuis* (virGp) are playing a major role in Glässer’s disease development in swine populations [15, 16]. However, other research studies demonstrated the role of non-virulent strains of *G. parasuis* (non-virGp) as an important colonizer to the swine nasal mucosa [17–19]. In connection with that, Bello-Ortí et al. [18] reported that non-virGp have the ability to form robust biofilms in contrast to virulent strains, and biofilm formation might therefore allow the non-virulent strains to colonize and persist in the upper respiratory tract of pigs. Thus, investigation of the polymicrobial interactions is essential to understand Glässer’s disease pathogenesis in a population.

Several studies have documented that the type of farm management practices and antibiotic use affect the incidence of swine respiratory diseases, including Glässer’s disease [20–22]. For instance, Hurnik et al. [23] reported several risk factors in the epidemiology of enzootic pneumonia such as feeding methods, housing type, farm size and management practices. As for the nasal microbial composition, Weese et al. [12] revealed that farm management practices can influence the nasal microbiota of pigs and subsequently the nasal methicillin-resistant *Staphylococcus aureus* carriage. More recently, Correa-Fiz et al. [1] concluded that the nasal microbiota composition was associated to the clinical status of the farm of origin of the piglets, leading to different susceptibilities to invasive infection by *G. parasuis*.

To the best of our knowledge, no study has investigated the association between the degree of virulence of *G. parasuis* strains and other members of the nasal microbiota. We hypothesized that members of the nasal microbiota associated with virGp might be different to the ones associated with non-virGp, establishing a differential network involving complex interactions. The results of this study will unravel the interaction of the nasal microbiota with *G. parasuis* strains, which will boost our understanding of the development of Glässer’s disease. The objective of this study is to investigate the statistical association patterns of nasal microbiome at different taxa levels (family and genus) with the virGp and non-virGp strains, while accounting for the farm management practices as a potential risk factors for the occurrence of Glässer’s disease in weaning piglets.

## Materials and methods

### Study population and sample collection

The study population, inclusion criteria and sample collection were fully described in Correa-Fiz et al. [1]. Briefly, DNA was extracted from nasal swabs of 51 randomly selected piglets at 3–4 weeks of age (at weaning). The microbiota composition was determined from sequenced amplicons spanning the V3–V4 region of the 16S rRNA gene generated by using the universal primers described previously by Klindworth et al. [24]. Seven swine farms located in Catalonia (Spain) were included in the study (Table 1). Four of these farms (MT, MC, RC, EJ) were having Glässer’s disease outbreaks (number of sampled piglets = 33), while the other three farms had no respiratory disease history (number of selected piglets = 18) and served as control farms (GM, PT, VL). Production system were a mix of multi-sites systems (*n* = 38 animals) in five farms (GM, PT, EJ, RC and MT) and farrow-to-finish systems (*n* = 13) in the other two farms (MC and VL).

**Table 1:** **Differentiation of** ***G. parasuis*** **strains into virulent and non-virulent by PCR assay and its frequency by farm, health status and production system type in 51 weaning piglets**

Item	Level	Virulent strains N (%)	Non-virulent strains N (%)	Total N (%)
Health status	Control (GM, PT, VL)	2 (8)	14 (52)	16 (31)
	Glässer’s disease (MT, MC, RC, EJ)	22 (92)	13 (48)	35 (69)
Production system type	Multi-sites (GM, PT, EJ, RC, MT)	24 (100)	24 (89)	48 (94)
	Farrow to finish (MC, VL)	0 (00)	3 (11)	3 (6)
Farm	EJ	10 (42)	0 (0)	10 (20)
	GM	2 (8)	9 (33)	11 (22)
	MC	0 (00)	0 (00)	0 (00)
	MT	6 (25)	6 (22)	12 (23)
	PRAT	0 (00)	2 (8)	2 (4)
	RC	6 (25)	7 (26)	13 (25)
	VL	0 (00)	3 (11)	3 (6)
Total		24 (47)	27 (53)	51 (100)

### DNA extraction and PCR assay

Total DNA was extracted from the nasal swabs using the Nucleospin Blood (Machinery Nagel) kit. Swabs were resuspended in PBS and suspensions were processed according to manufacturer’s instructions. *G. parasuis* strains were detected and differentiated into virulent and non-virulent using the specific PCR assay according to Galofré-Milà et al. [25].

### Statistical analysis

Data on nasal microbiota composition at the different taxonomical levels were available from Correa-Fiz et al. [1]. Prior to undertaking statistical analysis, data were screened for unlikely or missing values. No data were excluded on this basis. Subsequently, a descriptive statistical analysis was carried out to the potential risk factors related to piglets’ management (production system and health status) and nasal microbiota composition at family and genus levels based on the relative abundance of operational taxonomic units (OTUs).

We ran three different statistical models at family and genus levels of nasal microbiota including a multivariable logistic regression model with the virGp strain (present or absent) as the outcome variable and multivariable logistic regression with non-virGp strain (present or absent) as the outcome variable. The third model was a multivariable linear regression, which was built to investigate the association between nasal microbiota, and metadata with *G. parasuis* infection, as a continuous outcome variable. The outcome variable “*G. parasuis*” was skewed and was, therefore, transformed by taking their natural logarithm.

Initially, a univariable model was carried out to test the unconditional associations between dependent and various independent variables of interest including the potential farm factors and relative abundances of members of the nasal microbiota. Only independent variables with *P* ≤ 0.25 in this initial screening were included in multivariable logistic and linear regression models following the recommended strategy of Dohoo et al. [26]. Before proceeding with building our multivariable model analysis, we checked for correlations between the retained independent variables. If two variables were highly correlated (r > 0.8), only the one with the lower *P* value in the unconditional associations was retained. A multivariable model analysis including the significant variables from the univariable modelling was built using manual backward elimination procedure by dropping the least significant variable. The generated final model included only variables with *P* ≤ 0.05. Once the variables to be included in the final model were determined, two-way interactions were examined and retained if significant. The *P* value, odds ratio (OR) with a 95% confidence interval (95% CI), and regression coefficient (b) were recorded for each variable. In all statistical analyses, the results were considered to be significant at *P* ≤ 0.05. The models were constructed using the “*lm*” and “*glm*” functions for the linear and logistic regressions models, respectively in R version 3.3.3 [27].

## Results

### *Glaesserella parasuis* strains differentiation and distribution

Total DNA extracted from the nasal swabs collected from all the piglets under the study (*n* = 51) was used to determine the presence and the virulence of the *G. parasuis* strains. The proportion of virulent strains was 47% (24/51), whereas the proportion of non-virulent strains was 53% (27/51), Table 1. The virGp strains was the prevalent strain type in the Glässer’s disease group (43%, 22/51) in comparison to the control group. There was no difference between the proportion of the non-virGp strains in both Glässer’s disease and control groups. In respect to the type of production system, the proportion of the virGp and non-virGp strains was predominant in the multi-sites production systems (both of them 47%, 24/51) in comparison to farrow-to-finish system, Table 1. The farm EJ has the highest proportion of the virGp (20%, 10/51) over the other farms, whereas the farm GM showed the highest proportion of the non-virGp (18%, 9/51).

### Findings at family level

**Figure 1:**
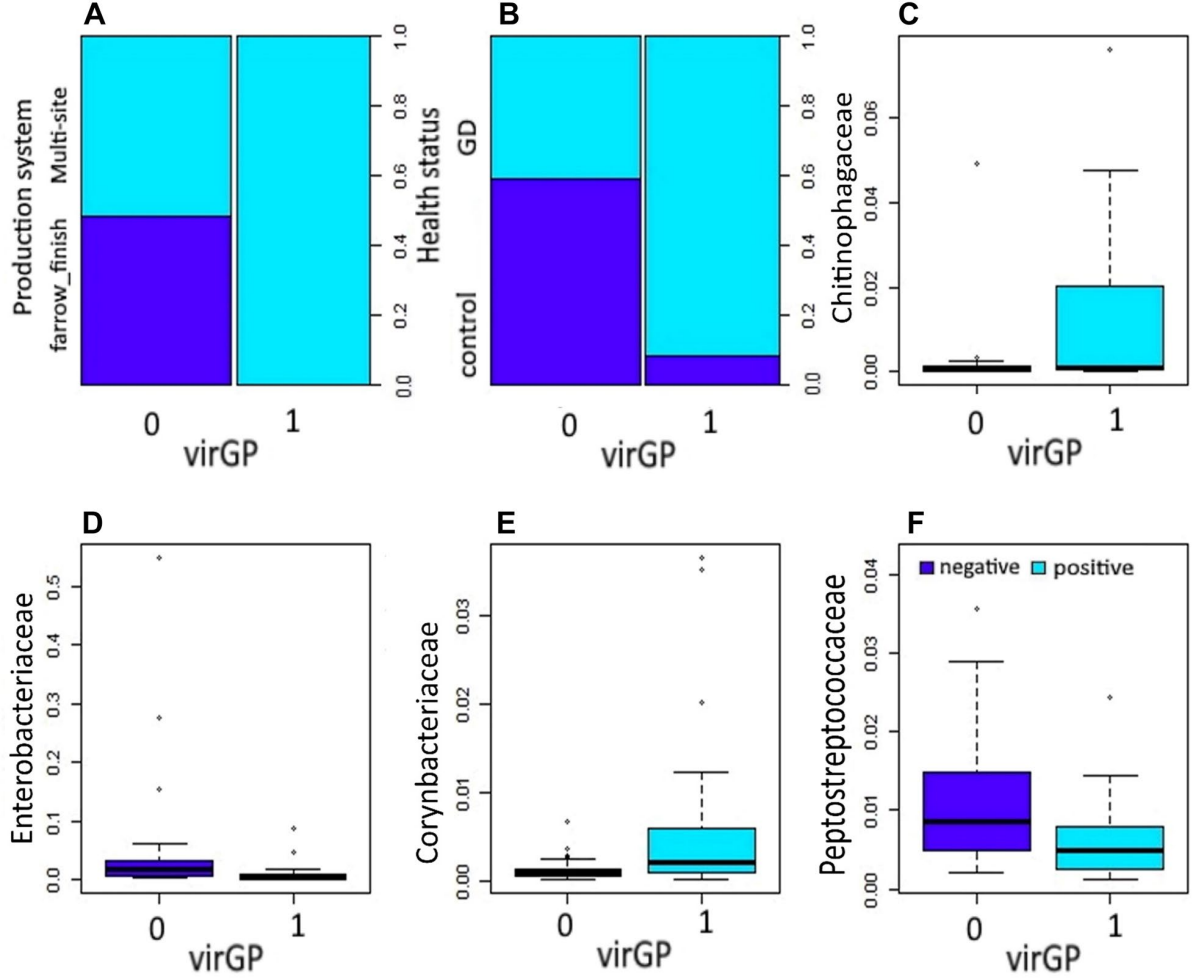
**Association of virulent** ***Glaesserella parasuis*** **strains (virGp) and significant bacterial families from the nasal microbiota.** Significant variables (*P* ≤ 0.05) influencing the colonization by virGp in 51 piglets found in the final model: production system (**A**), health status (**B**), and nasal microbiota members at family level *Chitinophagaceae* (**C**), *Enterobacteriaceae* (**D**), *Corynebacteriaceae* (**E**) and *Peptostreptococcaceae* (**F**).

**Figure 2:**
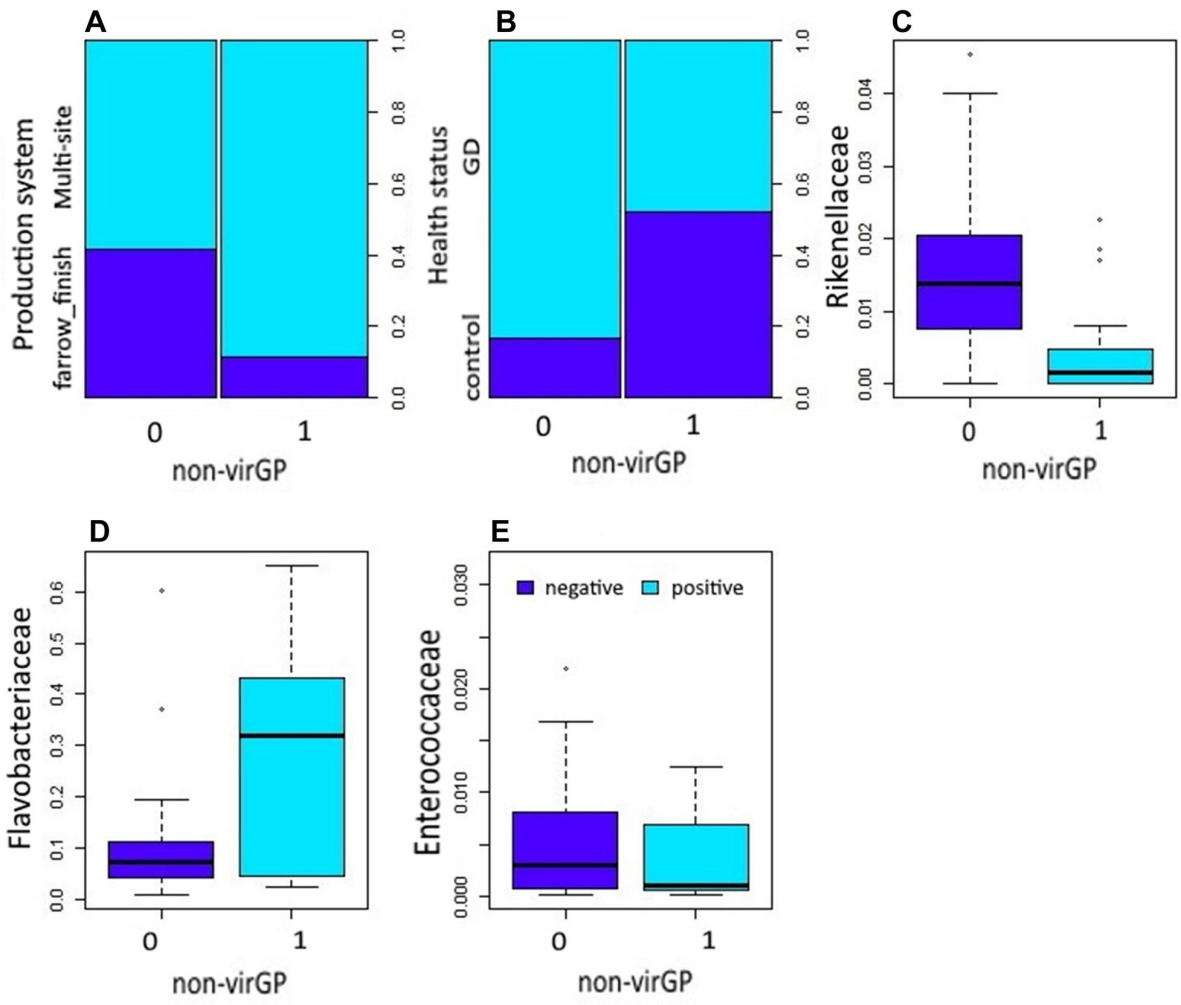
**Association of non-virulent** ***Glaesserella parasuis*** **strains (non-virGp) and significant bacterial families from the nasal microbiota.** Significant variables (*P* ≤ 0.05) influencing the colonization by non-virGp in 51 piglets found in the final model: production system (**A**), health status (**B**), and nasal microbiota members at family level *Rikenellaceae* (**C**), *Flavobacteriaceae* (**D**) and *Enterococcaceae* (**E**).

In total 284 bacterial families were revealed to colonize the nasal mucosa of piglets [1]. Out of this number, 52 families were identified as “others” and were discarded in this analysis. In total, 22 families were offered to statistical modelling after excluding 91 and 119 families with low relative abundance (cutoff < 0.01), and having few observations (≤ 10), respectively. The results of univariable model analyses of virulent strain, non-virulent strain, and relative abundance of *G. parasuis*, as natural logarithm at family level taxa are shown in Additional file 1. The results of final multivariable model analyses of virulent strain, non-virulent strain, and *G. parasuis* are shown in Tables 2, 3, and 4, respectively. The associations between the significantly variables presented in the final model and virulent strain, and non-virulent *G. parasuis* strains at family level are illustrated Figures 1 and 2, respectively. Illustration of the associations between the relative abundance of *G. parasuis*, expressed as natural logarithm in 51 piglets and significantly variables presented in the final model at family level is shown in Additional file 2.

**Table 2:** **Risk factors and nasal microbiota members (family level) associated with the presence of virulent strains of** ***G. parasuis*** **(*P* ≤ 0.05) in 51 piglets in 7 Spanish farms using a multivariable logistic regression model**

Variable	Level	Estimate	OR	95% CI	*P*
Production system	Farrow to finish	Ref	Ref	Ref	5.5e−07
	Multi-site	19.11	1.98	2.5e−46 to NA	
Health status	Control	Ref	Ref	Ref	6.4e−05
	Glässer’s disease	2.77	16.03	3.70–113.46	
*Chitinophagaceae*	–	250.9	8.8e+108	1.14e+30 to Inf	0.0102
*Enterobacteriaceae*	–	−51.7	3.5e−27	1.6e−62 to 1.9e−06	1.75e−05
*Corynebacteriaceae*	–	898.7	Inf	1.89e+135 to Inf	0.00022
*Peptostreptococcaceae*	–	−179.1	2.3e−74	1.8e−194 to 1.1e−10	0.017

Ref: reference, Inf: infinity.

**Table 3:** **Risk factors and nasal microbiota members (family level) associated with the presence of non-virulent strains of** ***G. parasuis*** **(*P* ≤ 0.05) in 51 piglets in 7 Spanish farms using a multivariable logistic regression model**

Variable	Level	Estimate	OR	95% CI	*P*
Production system	Farrow to finish	Ref	Ref	Ref	0.011
	Multi-site	−2.75	6.4e-02	3.01e−06 to 1.3e+02	
Health status	Control	Ref	Ref	Ref	0.0023
	Glässer’s disease	−6.12	2.2e−03	5.01e−07 to 1.2e−01	
*Rikenellaceae*	–	−597.77	2.5e−260	0.00 to 5.6e−72	1.6e−06
*Flavobacteriaceae*	–	11.67	1.2e+05	3.3e+01 to 7.3e+11	0.014
*Enterococcaceae*	–	−251.52	5.8e−110	2.8e−243 to 2.7e−27	0.0083

Ref: reference.

**Table 4:** **Risk factors and nasal microbiota members (family level) associated with relative abundance of** ***G. parasuis,*** **expressed as natural logarithm, (*P* ≤ 0.05) in 51 piglets in 7 Spanish farms using a multivariable linear regression model**

Family	Level	Estimate	95% CI	St. error	*P*
Production system	Farrow to finish	Ref	Ref	Ref	8.5e−08
	Multi-site	2.06	1.40–2.71	0.33	
Health status	Control	Ref	Ref	Ref	0.00514
	Glässer’s disease	0.80	0.20–1.40	0.30	
*Bacteroidaceae*	–	−38.1	(−61.3) to (−14.8)	11.6	4.03e−05
*Chitinophagaceae*	–	27.9	7.5–48.3	10.2	0.032
*Streptococcaceae*	–	13.9	4.9–22.9	4.5	0.004
*Mycoplasmataceae*	–	−5.8	(−11.2) to (−0.35)	2.7	0.037

Ref: reference.

### Microbial association with virulent strain of *G. parasuis*

Production system, health status, *Chitinophagaceae*, *Enterobacteriaceae*, *Corynebacteriaceae*, and *Peptostreptococcaceae* were significantly associated with the presence of virGp strains (Table 2). Piglets raised under the multi-sites production system showed a higher risk of having virGp strains by 1.98 compared to animals under the farrow to finish system. Piglets from farms with Glässer’s disease had higher odds of having virGp strain by 16.03 (95% CI 3.70–113.46) compared to animals from healthy farms. The presence of *Enterobacteriaceae*, and *Peptostreptococcaceae* in the nasal mucosa of piglets is associated with a low likelihood of colonization of virGp strain by −51.7 and −179.1, respectively. However, the presence of both *Chitinophagaceae*, and *Corynebacteriaceae* is associated with a higher probability of finding virGp strains by 250.9 and 898.7, respectively. A significant interaction was present between *Enterobacteriaceae* and *Corynebacteriaceae* indicating that both of them interfere significantly with colonization of virGp strain.

### Microbial association with non-virulent strain of *G. parasuis*

Production system, healthy status, *Rikenellaceae*, *Flavobacteriaceae*, and *Enterococcaceae* were significantly associated with the non-virGp strain (Table 3). Piglets raised under the multi-sites production system showed a lower risk of having non-virGp strains by 0.064 (95% CI 3.01e−06 to 1.3e+02) compared to animals under the farrow to finish system. Piglets from farms with Glässer’s disease had lower odds of having non-virGp strains by 0.0022 (95% CI 5.01e−07 to 1.2e−01) compared to piglets from healthy farms. Presence of *Rikenellaceae*, and *Enterococcaceae* in the nasal mucosa of piglets is associated with a low likelihood of colonization of non-virGp strain by −597.77 and −251.52, respectively. Whereas presence of *Flavobacteriaceae* is associated with a high likelihood of colonization of non-virGp strain by 11.67. A significant interaction was observed between *Rikenellaceae* and *Enterococcaceae* meaning that both of them interfere significantly with colonization of non-virGp strain.

### Microbial associations with *G. parasuis*

Production system, health status, *Bacteroidaceae*, *Chitinophagaceae*, *Streptococcaceae* and *Mycoplasmataceae* were significantly associated with *G. parasuis* abundance (Table 4). Piglets raised under the multi-sites production system were more likely to be colonized by *G. parasuis* by 2.06 (95% CI 1.40–2.71). Piglets from farms with Glässer’s disease were more likely to be colonized with *G. parasuis* by 0.80 (95% CI 0.20–1.40). Presence of *Bacteroidaceae*, and *Mycoplasmataceae* in the nasal mucosa of piglets is significantly associated with a low likelihood of *G. parasuis* colonization by −38.1 and −5.8, respectively. Whereas presence of *Chitinophagaceae* and *Streptococcaceae* is associated with a high likelihood of colonization of *G. parasuis* by 27.9 and 13.9. No significant interaction was detected.

### Findings at genus level

**Figure 3:**
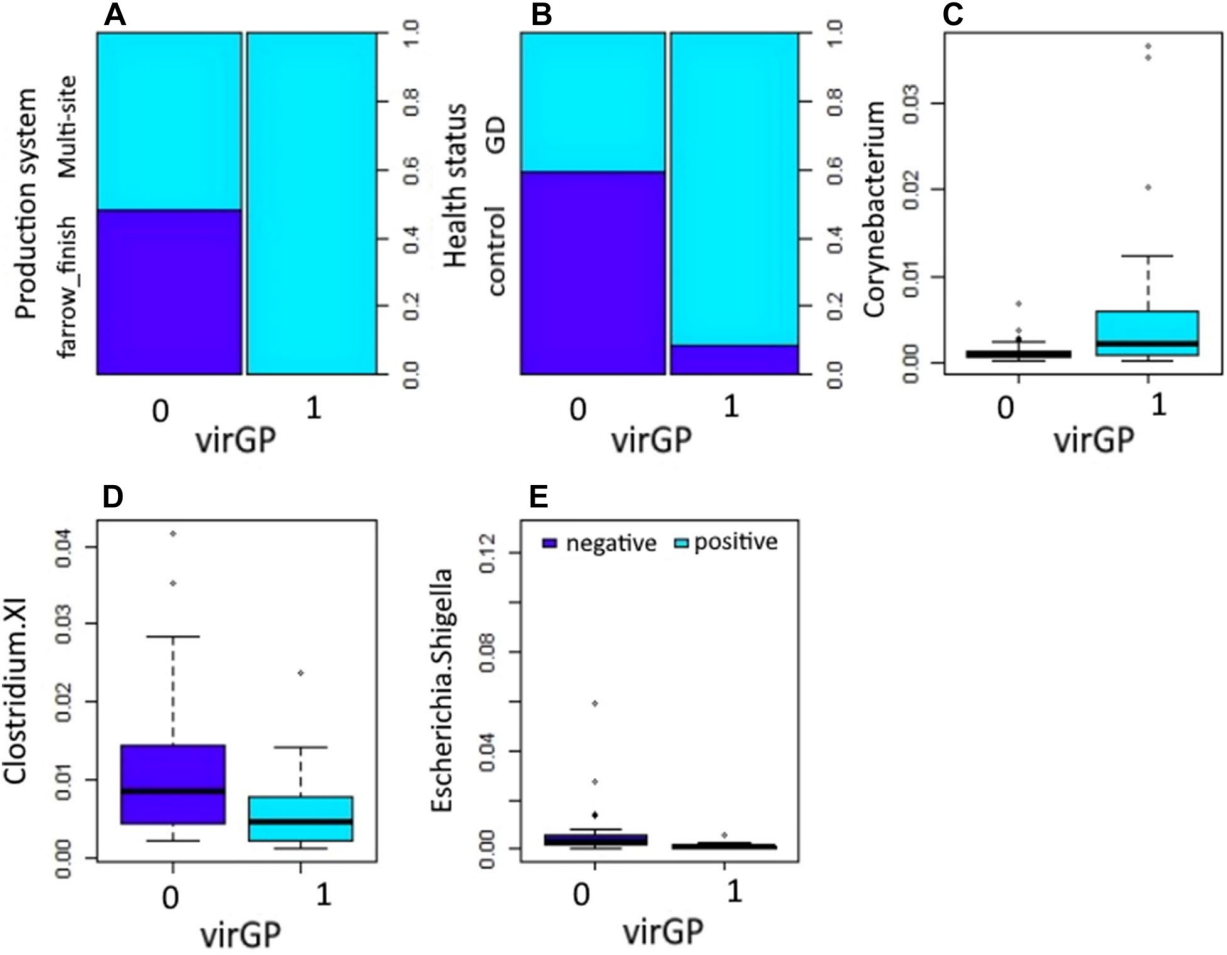
**Association of virulent** ***Glaesserella parasuis*** **strains (virGp) and significant bacterial genera from the nasal microbiota.** Significant variables (*P* ≤ 0.05) influencing the colonization by virGp in 51 piglets found in the final model: production system (**A**), health status (**B**), and nasal microbiota members at genus level *Corynebacterium* (**C**), *Clostridium XI* (**D**) and *Escherichia.Shigella* (**E**).

**Figure 4:**
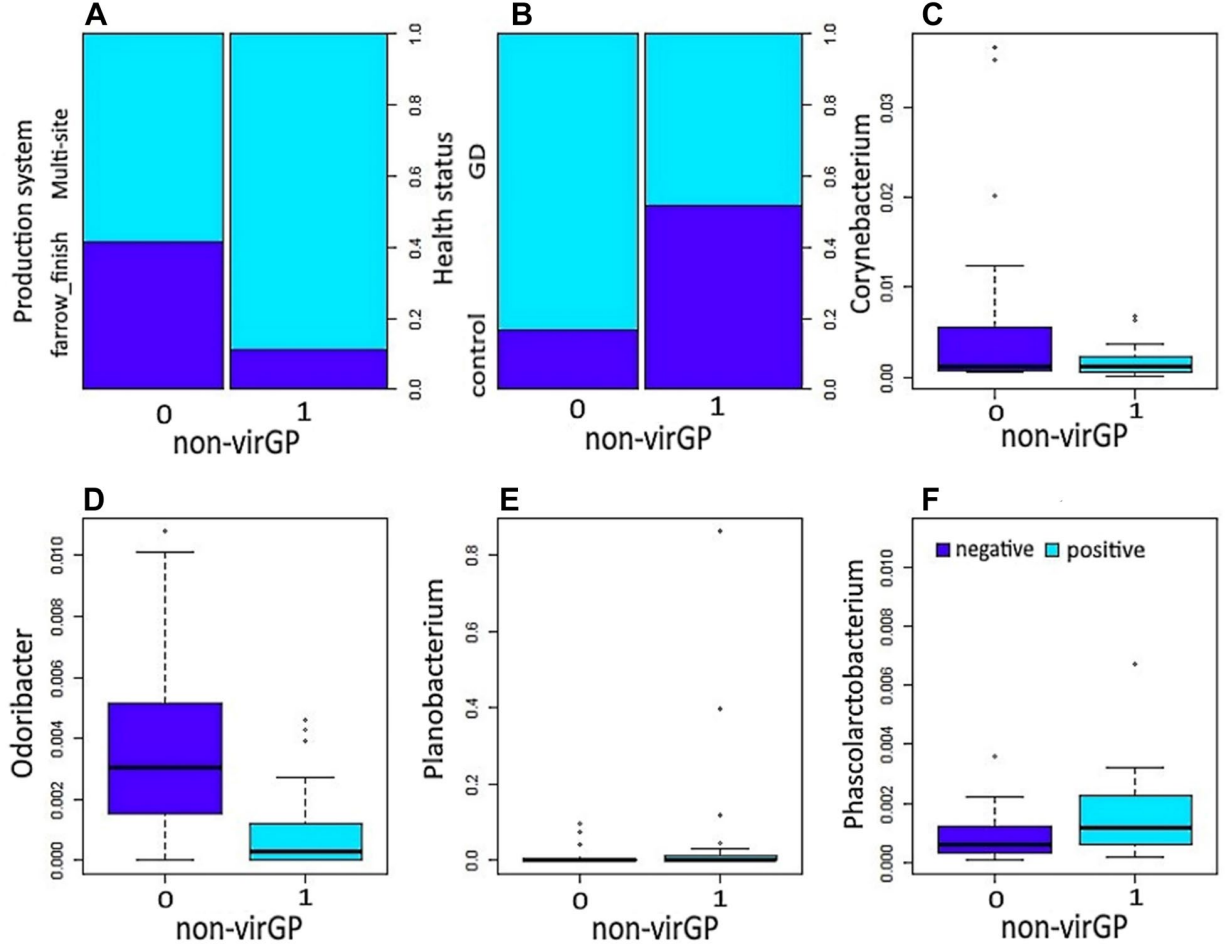
**Association of non-virulent** ***Glaesserella parasuis*** **strains (non-virGp) and significant bacterial genera from the nasal microbiota.** Significant variables (*P* ≤ 0.05) influencing the colonization of non-virGp in 51 piglets found in the final model: production system (**A**), health status (**B**), and nasal microbiota members at genus level *Corynebacterium* (**C**), *Odoribacter* (**D**), *Planobacterium* (**E**) and *Phascolarctobacterium* (**F**).

In total, we got 949 bacterial genera colonizing the nasal mucosa. Out of this number, 154 genera were identified as others/unassigned/unclassified and were discarded. In total, 45 genera were used in a valid analysis after excluding 251 and 499 genera with low abundance (cutoff ≤ 0.01), and having few observations (≤ 10), respectively. The results of univariable model analyses of virulent strain, non-virulent strain, and relative abundance of *G. parasuis*, as natural logarithm at genus level taxa are shown in Additional file 3. The results of final multivariable analyses of virulent, non-virulent strains, and *G. parasuis* are presented in Tables 5, 6, and 7, respectively. The associations between the significantly variables presented in the final model and virulent strain, and non-virulent *G. parasuis* strains at genus level are illustrated Figures 3 and 4, respectively. Illustration of the associations between the relative abundance of *G. parasuis*, expressed as natural logarithm in 51 piglets and significantly variables presented in the final model at genus level is shown in Additional file 2.

**Table 5:** **Risk factors and nasal microbiota members (genus level) associated with the presence of virulent strains of** ***G. parasuis*** **(*P* ≤ 0.05) in 51 piglets in 7 Spanish farms using a multivariable logistic regression model**

Genus/variable	Level	Estimates	OR	%95 CI	*P*
Health status	Control	Ref	Ref	Ref	6.48e−05
	Glässer’s disease	3.69	4.03+01	7.1 to 3.7e+02	
Production system	Farrow to finish	Ref	Ref	Ref	5.56e−07
	Multi-site	20.70	9.8+08	1.1e−73 to Inf	
*Corynebacterium*	–	674.01	5.3e+292	7.03e+61 to Inf	0.0013
*Clostridium.XI*	–	−204.3	1.9e−89	1.8e−200 to 1.03e−15	5.5e−05
*Escherichia.Shigella*	–	−295.6	4.4e−129	0.00 to 3.8e−01	0.0394

Ref: reference, Inf: infinity.

**Table 6:** **Risk factors and nasal microbiota members (genus level) associated with the presence of non-virulent strains of** ***G. parasuis*** **(*P* ≤ 0.05) in 51 piglets in 7 Spanish farms using a multivariable logistic regression model**

Genus/variable	Level	Estimates	OR	%95 CI	*P*
Health status	Control	Ref	Ref	Ref	0.0073
	Glässer’s disease	−2.22	0.11	0.015–0.48	
Production system	Farrow to finish	Ref	Ref	Ref	0.0034
	Multi-site	2.34	10.34	2.045–86.57	
*Corynebacterium*	–	−116.6	2.4-51	6.4e−220 to 1.9e+10	0.0134
*Odoribacter*	–	−929.7	0.00	0.00 to 1.1e−187	5.8e−05
*Planobacterium*	–	17.06	2.6e+07	2.2 to 4.5e+27	0.037
*Phascolarctobacterium*	–	518.5	1.6e+225	5.1e+12 to Inf	0.034

Ref: reference, Inf: infinity.

**Table 7:** **Risk factors and nasal microbiota members (genus level) associated with relative abundance of** ***G. parasuis,*** **expressed as natural logarithm, (*P* ≤ 0.05) in 51 piglets in 7 Spanish farms using a multivariable linear regression model**

Genus	Level	Coff.	95% CI	St. Error	*P*
Health status	Control	Ref	Ref	Ref	0.00514
	Glässer’s disease	0.798	0.20–1.40	0.297	
Production system	Farrow to finish	Ref	Ref	Ref	8.5e−08
	Multi-site	2.054	1.40–2.71	0.326	
*Alloprevotella*	–	33.1	−34.9 to 101.1	33.7	0.0019
*Streptococcus*	–	8.1	0.45–15.8	3.8	0.0054
*Clostridium.XI*	–	4.9	−27.9 to 37.8	16.3	0.055
*Oscillibacter*	–	−82.6	−125.9 to (−39.3)	21.5	6.2e−06
*Kingella*	–	53.5	13.5–93.4	19.8	0.012
*Actinobacillus*	–	27.1	14.3–39.9	6.4	0.0001

Ref: reference.

### Microbial associations with virulent strain of *G. parasuis*

Production system, health status, *Corynebacterium*, *Clostridium XI*, and *Escherichia/Shigella* were significantly associated with the virGp strain as depicted in Table 5. Piglets raised under the multi-sites production system were more likely to be have a higher risk of virGp strain by 9.8+08 compared to animals under the farrow to finish system. Piglets with Glässer’s disease were more likely to harbor virGp strain by 4.03+01 (95% CI 7.1 to 3.7e+02) compared to non-infected animals. Presence of *Clostridium XI*, and *Escherichia/Shigella* in the nasal mucosa of piglets reduce the colonization of virGp strain by −204.3 and −295.6, respectively. Whereas presence of *Corynebacterium* increases the colonization of virGp strain by 674.01. A significant interaction was present between *Corynebacterium* and *Escherichia/Shigella*.

### Microbial associations with non-virulent strain of *G. parasuis*

Production system, health status, *Corynebacterium*, *Odoribacter*, *Planobacterium* and *Phascolarctobacterium* were significantly associated with the non-virulent strain of *G. parasuis* (Table 6). Piglets raised under the multi-sites production system have a higher odd of non-virGp strains by 10.34 (95% CI 2.045–86.57) compared to animals under the farrow to finish system. Piglets with Glässer’s disease have lower odds of virGp strain by 0.11 (95% CI 0.015–0.48) compared to healthy animals. Presence of *Corynebacterium*, and *Odoribacter* in the nasal mucosa of piglets is associated with a low likelihood of colonization of non-virGp strains by −116.6 and −929.7, respectively. Whereas the presence of *Planobacterium* and *Phascolarctobacterium* is associated with a high likelihood of colonization of non-virulent strains by 17.06 and 518.5. A significant interaction was present between *Odoribacter* and *Phascolarctobacterium.*

### Microbial association with *G. parasuis*

Production system, health status, *Alloprevotella*, *Streptococcus*, *Clostridium XI*, *Oscillibacter*, *Kingella*, and *Actinobacillus* were significantly associated with *G. parasuis* (Table 7). Piglets raised under the multi-sites production system are more susceptible to *G. parasuis* by 2.054 (95% CI 1.40–2.71). Piglets with Glässer’s disease have a higher odds *G. parasuis* by 0.80 (95% CI 0.20–1.40). Presence of *Oscillibacter* in the nasal mucosa of piglets is significantly associated with a low likelihood of colonization of *G. parasuis* by −82.6. Whereas presence of *Alloprevotella*, *Streptococcus*, *Clostridium XI*, *Kingella*, and *Actinobacillus* is associated with a high likelihood of colonization of *G. parasuis* by 33.1, 8.1, 4.9, 53.5, and 27.1. A significant interaction was present between *Alloprevotella* and *Oscillibacter*, as well as between *Streptococcus* and *Oscillibacter.*

## Discussion

### Factors affecting Glässer’s disease epidemiology

Our findings demonstrated that the type of porcine production system has a significant impact on the epidemiology of Glässer’s disease. We found that the multi-site production type increases the prevalence of the virGp strains and subsequently, increases the risk of Glässer’s disease in comparison to the one site production type (farrow-to-finish). In agreement with that, Rapp-Gabrielson et al. [4] reported that adoption of new production systems, particularly multiple site production systems based on a single origin of the pigs, together with the emergence of respiratory and systemic syndromes have contributed to an increase in prevalence and severity of Glässer’s disease. Plausibly, this finding could be argued by the stress factors caused by the additional animal handling and transportation. The relocation of the weaning piglets from one environment and management conditions to another can cause a suppression of the animal immune system [28] and subsequently might favor the development of Glässer’s disease. This is consistent with the conclusion of Nielsen and Danielson [29], who demonstrated that the immune status of the herd is a determinant of the pathogenic outcome of the infection. Nevertheless, the heterogeneity in virulence among *G. parasuis* strains could also be a significant determinant for disease development [30, 31]. In line with that, Aragon et al. [30] stated that basically all piglets are colonized by *G. parasuis* but only specific strains are capable of inducing the clinical form of Glässer’s disease, sometimes following a perturbation of their host due to distress factors and/or co-infections.

Piglets from farms with Glässer’s disease have higher odds of being colonized by virGp strains than those from healthy farms. This finding strongly reflects that the virulence of the *G. parasuis* strains is playing the major role in Glässer’s disease development and epidemiology. The reported findings in this study are also in agreement with previous studies demonstrating a marked difference between the epidemiological, molecular and virulence patterns, and clinical manifestations associated with different strains of *G. parasuis* [3, 15, 25, 32, 33]. From the epidemiological and microbial point of view, we have shown that the identification and differentiation of *G. parasuis* at the strain level could be used for prognosis of Glässer’s disease in swine farms.

### Microbiota associated with *G. parasuis* of different virulence

A strong association pattern was reported between the microbiota with the virGp and non-virGp strains at different taxa levels. In that respect it was reported that some microbial families could nourish the colonization and presence of the *G. parasuis* strains, while other different microbial families suppress/compete its colonization in the environment [1]. One theory that could explain the reported finding is the antagonistic and synergistic effects of the microbial populations, which inhabiting together under the same environmental conditions. We found that *Streptococcus*, *Clostridium XI*, *Kingella*, and *Actinobacillus* increases the presence of *G. parasuis*. This is in agreement with the findings of previous studies [1, 34, 35]. Slifierz et al. [35] found that the most common core OTUs (> 1% relative abundance in ≥ 80% of pigs) prior to weaning were from *Clostridia*, while Correa-Fiz et al. [1] reported a higher relative abundance of *Mycoplasmataceae*, *Streptococcus* and *Haemophilus* in swine farms with Glässer’s disease. We found that *Mycoplasmataceae* showed a significant reduction in the likelihood of colonization of *G. parasuis* by −5.8, whereas *Streptococcaceae* increase the likelihood of colonization of *G. parasuis* by 13.9. This shows that different bacterial species pose different association patterns with *G. parasuis*. Schmidt et al. [34] demonstrated that the *Peptostreptococcaceae* family was one of the most abundant members of the *Clostridia* class colonizing the piglet gut. The connection between the microbial communities of gut and the respiratory system has been reported [36]. Here, the families *Enterobacteriaceae* and *Peptostreptococcaceae* significantly reduced the likelihood of colonization of virGp strain by −51.7 and −179.1. Additionally, previous reports showed that gut of neonates is initially colonized by members of *Enterobacteriaceae* and *Streptococcaceae* [37, 38]. This interesting finding could shed the light on the role of *Peptostreptococcaceae*, *Enterobacteriaceae* and *Streptococcaceae* in the control of Glässer’s disease in piglets.

Interestingly, those families and genera significantly associated with virGp and non-virGp strains were different from those taxa that were significantly associated with *Glaesserella* genus (as outcome), whose only known species in swine is *G. parasuis*. These discrepancies in the families and genera associated with the two types of strains and *G. parasuis* are not only important for understanding Glässer’s disease biology but also for ascertaining our needs for accurate and precise diagnosis at strain level. It seems that some microbial populations, from *Chitinophagaceae* and *Corynebacteriaceae* families, may facilitate the colonization of virGp strains, meanwhile members from the *Flavobacteriaceae* family could facilitate the colonization of non-virGp strains. On the other hand, families such as *Enterobacteriaceae* and *Peptostreptococcaceae* may act as inhibitors of the virGp strains, whereas *Rikenellaceae*, and *Enterococcaceae* could have an inhibitory effect on the non-virGp strains. The variations in the microbiota composition associated with both types of *G. parasuis* strains could be driven by the physiology of the different bacterial community, which could impact the magnitude of virulent or non-virulent strains colonization and prevalence, and subsequently, the piglet’s health status [4]. In a similar infection study, Espinosa-Gongora et al. [13] reported that nasal microbiome of pigs that are not colonized with *Staphylococcus aureus* harbors several species/taxa that are significantly less abundant in carrier pig, suggesting that the nasal microbiota plays a role in the individual predisposition to *S. aureus* nasal carriage in pigs. In the earlier study by Correa-Fiz et al. [1], a marked difference between the composition and diversity of the nasal microbiota between swine farms with different health status was reported. The authors added that interactions between the host and microbial communities of nasal mucosa may result in selection of the beneficial bacteria, which can prevent the colonization by pathogens, and that could be the case for the non-virGp strains. We have elucidated that hypothesis at strain level for *G. parasuis* showing that there are wide variations in the association of microbial communities with different type of strains. Several genera were found to be associated to strains of *G. parasuis* depending on their virulence. For instance, the presence of *Escherichia/Shigella* reduces the probability of colonization by virGp, while the presence of *Phascolarctobacterium* is associated with a high likelihood of colonization by non-virGp. These two genera from the nasal microbiota of piglets have been recently found to be associated to a healthier farm status [39] and deserve further investigation on their role in Glässer’s disease. To the best of our knowledge, this is the first study investigating microbial associations with virulent and non-virulent strains of *G. parasuis*. Previous research hypothesized that the increased abundance and/or prevalence of certain pathogens in the microbial environment could confer a higher risk to develop specific disease [1, 40]. The reported findings in this study strongly and significantly stand with this hypothesis for Glässer’s disease development in swine populations.

One limitation of our study is the sample size of the study group. In any observational epidemiological studies, we collect data on a subset of animals (study group) in the source study population of interest, which may result in a random error bias due to low sample size. However, Dohoo [41] stated that even if this subset is a true random sample, the obtained estimates will vary somewhat from the true population value as a result of random variation inherent in the sampling process. However, one way to address this uncertainty in the estimate is by computing a confidence interval [41], which provides the range of values within which the true population value might lie as we have reported in our findings.

In terms of the applicability and generalizability of the obtained findings, the selection criteria for the study populations should be taken in mind. Particularly, it is well-established that the microbiota composition is dynamically changed and influenced by many contributing factors, including day of sampling, feeding management, breed, age, and health status.

Gaining knowledge on the polymicrobial interactions could provide new insights into the disease pathogenesis, as well as novel avenues for prevention and control of pathogenic infections. Although biological validation of our statistical findings is needed, the marked variation in the associations of different microbial taxa inhabiting the swine nasal niche with the colonization of virGp and non-virGp strains points to new possibilities in the manipulation of the microbiota composition to reduce the colonization by this pathogen.

The multi-site production system and disease status of the farm were both significantly associated with the presence of virulent strains. Interestingly, we found a wide variation in the association of nasal microbiota communities with virGp and non-virGp strains in weaning piglets. The findings of this study boost our understanding of Glässer’s disease development and could be a base for innovative non-antimicrobial alternatives for Glässer’s disease control.

## Supplementary information

**Additional file 1. The results of univariable model analyses of virulent strains (A), non-virulent strains (B), and relative abundance of** ***G. parasuis*** **(C), expressed as natural logarithm at family level taxa. A** Univariable logistic regression for potential risk factors and nasal microbiota at family level associated with the presence of virulent strains of *G. parasuis* (virGp) infection (*P*-value ≤ 0.25) in 51 piglets in 7 Spanish farms. **B** Univariable logistic regression for potential risk factors and nasal microbiota at family level associated with the presence of non-virulent strains of *G. parasuis* (non-virGp) infection (*P*-value ≤ 0.25) in 51 piglets in 7 Spanish farms. **C** Univariable linear regression for potential risk factors and nasal microbiota at family level associated with relative abundance of *G. parasuis*, expressed as natural logarithm, (*P* ≤ 0.25) in 51 piglets in 7 Spanish farms.

**Additional file 2. Illustration of the associations between the relative abundance of** ***Glaesserella parasuis*, expressed as natural logarithm in 51 piglets and significantly variables presented in the final model including farm management factors (production system and health status) and nasal microbiota members at family (A) and genus (B) taxa. A** Plotting the association between the relative abundance of *Glaesserella parasuis*, expressed as natural logarithm in 51 piglets and significantly variables (*P* ≤ 0.05) presented in the final model including farm management factors (production system and health status) and nasal microbiota members at family level (*Bacteroidaceae*, *Chitinophagaceae*, *Streptococcaceae,* and *Mycoplasmataceae*). **B** Plotting the association between the relative abundance of *Glaesserella parasuis*, expressed as natural logarithm in 51 piglets and significantly variables (*P* ≤ 0.05) presented in the final model including farm management factors (production system and health status) and nasal microbiota members at genus level (*Alloprevotella*, *Streptococcus*, *Clostridium XI*, *Oscillibacter*, *Kingella* and *Actinobacillus*).

**Additional file 3. The results of univariable model analyses of virulent strain (A), non-virulent strain (B), and relative abundance of** ***G. parasuis*** **(C), expressed as natural logarithm at genus level taxa. A** Univariable logistic regression for potential risk factors and nasal microbiota at genus level associated with the virulent strain of *G. parasuis* (virGp) infection (*P*-value ≤ 0.25) in 51 piglets in 7 Spanish farms. **B** Univariable logistic regression for potential risk factors and nasal microbiota at genus level associated with the non-virulent strain of *G. parasuis* (non-virGp) infection (*P*-value ≤ 0.25) in 51 piglets in 7 Spanish farms. **C** Univariable linear regression for potential risk factors and nasal microbiota at genus level associated with relative abundance of *G. parasuis*, expressed as natural logarithm, (*P*-value ≤ 0.25) in 51 piglets in 7 Spanish farms.

## Data Availability

The datasets of the current study are available from the corresponding author on reasonable request.
