# Supplementary material for: Variations in association of nasal microbiota with virulent and non-virulent strains of Glaesserella (Haemophilus) parasuis in weaning piglets
Source: Vet Res. 2020 Feb 3;51:7. doi: 10.1186/s13567-020-0738-8 (PMC6996185; doi:10.1186/s13567-020-0738-8)
Supplement: Supplementary file 1 — Additional file 1. The results of univariable model analyses of virulent strains (A), non-virulent strains (B), and relative abundance of G. parasuis (C), expressed as natural logarithm at family level taxa. A Univariable logistic regression for potential risk factors and nasal microbiota at family level associated with the presence of virulent strains of G. parasuis (virGp) infection (P-value ≤ 0.25) in 51 piglets in 7 Spanish farms. B Univariable logistic regression for potential risk factors and nasal microbiota at family level associated with the presence of non-virulent strains of G. parasuis (non-virGp) infection (P-value ≤ 0.25) in 51 piglets in 7 Spanish farms. C Univariable linear regression for potential risk factors and nasal microbiota at family level associated with relative abundance of G. parasuis, expressed as natural logarithm, (P ≤ 0.25) in 51 piglets in 7 Spanish farms. [file 13567_2020_738_MOESM1_ESM.docx]

**Additional file 1** **The results of univariable model analyses of virulent strain (Additional file 1A), non-virulent strain (Additional file 1B), and relative abundance of *H. parasuis* (Additional file 1C), expressed as natural logarithm at family level taxa.**

**Additional file 1A Univariable logistic regression for potential risk factors and nasal microbiota at family level associated with the presence of virulent strains of *H. parasuis* infection (*P-*value ≤ 0.25) in 51 piglets in 7 Spanish farms.**

| **Variable** | **Level** | **Estimate** | ***P* value** |
| --- | --- | --- | --- |
| Production system | Farrow to finish | Ref | 5.938e-06 |
|  | Multi-site | 19.11 |  |
| Health status | Control | Ref | 6.481e-05 |
|  | Glässer’s disease | 2.773 |  |
| *Micrococcaceae* | --- | 31.4784 | 0.4709 |
| *Bacteroidaceae* | --- | -1.49622 | 0.9399 |
| *Porphyromonadaceae* | --- | 6.2370 | 0.5816 |
| *Rikenellaceae* | --- | -0.04707 | 0.9985 |
| *Chitinophagaceae* | --- | 57.2805 | 0.01017 |
| *Staphylococcaceae* | --- | 14.4917 | 0.4739 |
| *Lactobacillaceae* | --- | 3.4791 | 0.8536 |
| *Streptococcaceae* | --- | 7.3267 | 0.3744 |
| *Ruminococcaceae* | --- | -17.4475 | 0.02425 |
| *Campylobacteraceae* | --- | 106.3968 | 0.2957 |
| *Enterobacteriaceae* | --- | -34.1691 | 0.008074 |
| *Corynebacteriaceae* | --- | 349.6166 | 0.001306 |
| *Prevotellaceae* | --- | -7.1436 | 0.5252 |
| *Flavobacteriaceae* | --- | 1.5493 | 0.3079 |
| *Enterococcaceae* | --- | 33.396 | 0.47 |
| *Clostridiaceae1* | --- | -127.6040 | 0.006036 |
| *Lachnospiraceae* | --- | -3.9730 | 0.4169 |
| *Peptostreptococcaceae* | --- | -120.7150 | 0.005894 |
| *Neisseriaceae* | --- | 3.5826 | 0.7847 |
| *Moraxellaceae* | --- | -1.6695 | 0.3634 |
| *Mycoplasmataceae* | --- | -2.23894 | 0.6581 |

**Additional file 1B** **Univariable logistic regression for potential risk factors and nasal microbiota at family level associated with the presence of non-virulent strains of *H. parasuis* infection (*P-*value ≤ 0.25) in 51 piglets in 7 Spanish farms.**

| Variable | Level | Estimate | *P* value |
| --- | --- | --- | --- |
| Production system | Farrow to finish | Ref | 0.01102 |
|  | Multi-sites | 1.7430 |  |
| Health status | Control | Ref | 0.007276 |
|  | Glässer’s disease | -1.6835 |  |
| *Micrococcaceae* | --- | -82.0700 | 0.08338 |
| *Bacteroidaceae* | --- | -91.1895 | 0.0003249 |
| *Porphyromonadaceae* | --- | -50.1300 | 0.0006415 |
| *Rikenellaceae* | --- | -169.1647 | 1.082e-05 |
| *Chitinophagaceae* | --- | 26.08052 | 0.1662 |
| *Staphylococcaceae* | --- | 6.4271 | 0.7465 |
| *Lactobacillaceae* | --- | 5.31128 | 0.7795 |
| *Streptococcaceae* | --- | -6.6956 | 0.4153 |
| *Ruminococcaceae* | --- | -10.3546 | 0.1601 |
| *Campylobacteraceae* | --- | 119.855701 | 0.2953 |
| *Enterobacteriaceae* | --- | -2.7116 | 0.4397 |
| *Corynebacteriaceae* | --- | -178.2543 | 0.01338 |
| *Prevotellaceae* | --- | 3.20032 | 0.7721 |
| *Flavobacteriaceae* | --- | 5.8083 | 0.001144 |
| *Enterococcaceae* | --- | -96.4277 | 0.06297 |
| *Clostridiaceae1* | --- | -8.8473 | 0.813 |
| *Lachnospiraceae* | --- | -17.7080 | 0.001595 |
| *Peptostreptococcaceae* | --- | 4.16895 | 0.8982 |
| *Neisseriaceae* | --- | 18.9379 | 0.1858 |
| *Moraxellaceae* | --- | 0.58198 | 0.7486 |
| *Mycoplasmataceae* | --- | -10.6057 | 0.06061 |

**Additional file 1C Univariable linear regression for potential risk factors and nasal microbiota at family level associated with relative abundance of *H. parasuis*, expressed as natural logarithm, (*P* ≤ 0.25) in 51 piglets in 7 Spanish farms.**

| Variable | Level | Estimate | St. Error | *P* value |
| --- | --- | --- | --- | --- |
| Production system | Farrow to finish | Ref | Ref | 2.221e-07 |
|  | Multi-sites | 2.0439 | 0.3398 |  |
| Health status | Control | Ref | Ref | 0.09624 |
|  | Glässer’s disease | 0.6735 | 0.3971 |  |
| *Micrococcaceae* | --- | -17.3432 | 29.9660 | 0.5654 |
| *Bacteroidaceae* | --- | -46.7409 | 12.0830 | 0.0003236 |
| *Porphyromonadaceae* | --- | -22.6343 | 7.1608 | 0.002697 |
| *Rikenellaceae* | --- | -59.7156 | 15.3913 | 0.0003121 |
| *Chitinophagaceae* | --- | 31.2755 | 10.9507 | 0.006276 |
| *Staphylococcaceae* | --- | 0.9453 | 13.5963 | 0.9449 |
| *Lactobacillaceae* | --- | -19.0136 | 12.8553 | 0.1455 |
| *Streptococcaceae* | --- | 8.5570 | 5.5150 | 0.1272 |
| *Ruminococcaceae* | --- | -10.744 | 4.790 | 0.02946 |
| *Campylobacteraceae* | --- | 48.8115 | 54.4409 | 0.3743 |
| *Enterobacteriaceae* | --- | -3.5102 | 2.2353 | 0.1228 |
| *Corynebacteriaceae* | --- | -12.2797 | 26.5913 | 0.6463 |
| *Prevotellaceae* | --- | -1.9647 | 7.6312 | 0.7979 |
| *Flavobacteriaceae* | --- | 1.4948 | 1.0274 | 0.1521 |
| *Enterococcaceae* | --- | 11.0567 | 31.6669 | 0.7285 |
| *Clostridiaceae.1* | --- | -6.7464 | 26.0337 | 0.7966 |
| *Lachnospiraceae* | --- | -8.9537 | 3.1038 | 0.005807 |
| *Peptostreptococcaceae* | --- | -31.8789 | 22.1687 | 0.1568 |
| *Neisseriaceae* | --- | 3.8648 | 9.1141 | 0.6734 |
| *Moraxellaceae* | --- | -1.3609 | 1.2460 | 0.2801 |
| *Mycoplasmataceae* | --- | -6.1981 | 3.3589 | 0.07105 |
