# Supplementary material for: Variations in association of nasal microbiota with virulent and non-virulent strains of Glaesserella (Haemophilus) parasuis in weaning piglets
Source: Vet Res. 2020 Feb 3;51:7. doi: 10.1186/s13567-020-0738-8 (PMC6996185; doi:10.1186/s13567-020-0738-8)
Supplement: Supplementary file 2 — Additional file 2. Illustration of the associations between the relative abundance of Glaesserella parasuis, expressed as natural logarithm in 51 piglets and significantly variables presented in the final model including farm management factors (production system and health status) and nasal microbiota members at family (A) and genus (B) taxa. A Plotting the association between the relative abundance of Glaesserella parasuis, expressed as natural logarithm in 51 piglets and significantly variables (P ≤ 0.05) presented in the final model including farm management factors (production system and health status) and nasal microbiota members at family level (Bacteroidaceae, Chitinophagaceae, Streptococcaceae, and Mycoplasmataceae). B Plotting the association between the relative abundance of Glaesserella parasuis, expressed as natural logarithm in 51 piglets and significantly variables (P ≤ 0.05) presented in the final model including farm management factors (production system and health status) and nasal microbiota members at genus level (Alloprevotella, Streptococcus, Clostridium XI, Oscillibacter, Kingella and Actinobacillus). [file 13567_2020_738_MOESM2_ESM.docx]

**Additional file 2** **Illustration of the associations between the relative abundance of *Glaesserella parasuis*, expressed as natural logarithm in 51 piglets and significantly variables presented in the final model including farm management factors (production system and health status) and nasal microbiota members at family (Additional file 2A) and genus (Additional file 2B) taxa.**


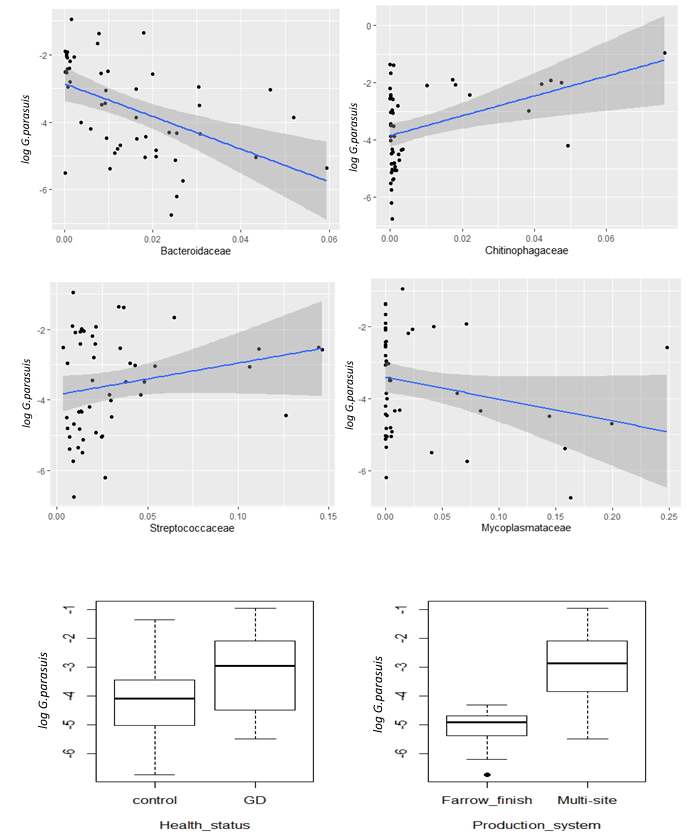


Additional file 2A Plotting the association between the relative abundance of *Glaesserella parasuis*, expressed as natural logarithm in 51 piglets and significantly variables (*P* ≤ 0.05) presented in the final model including farm management factors (production system and health status) and nasal microbiota members at family level (*Bacteroidaceae*, *Chitinophagaceae*, *Streptococcaceae*, and *Mycoplasmataceae*).


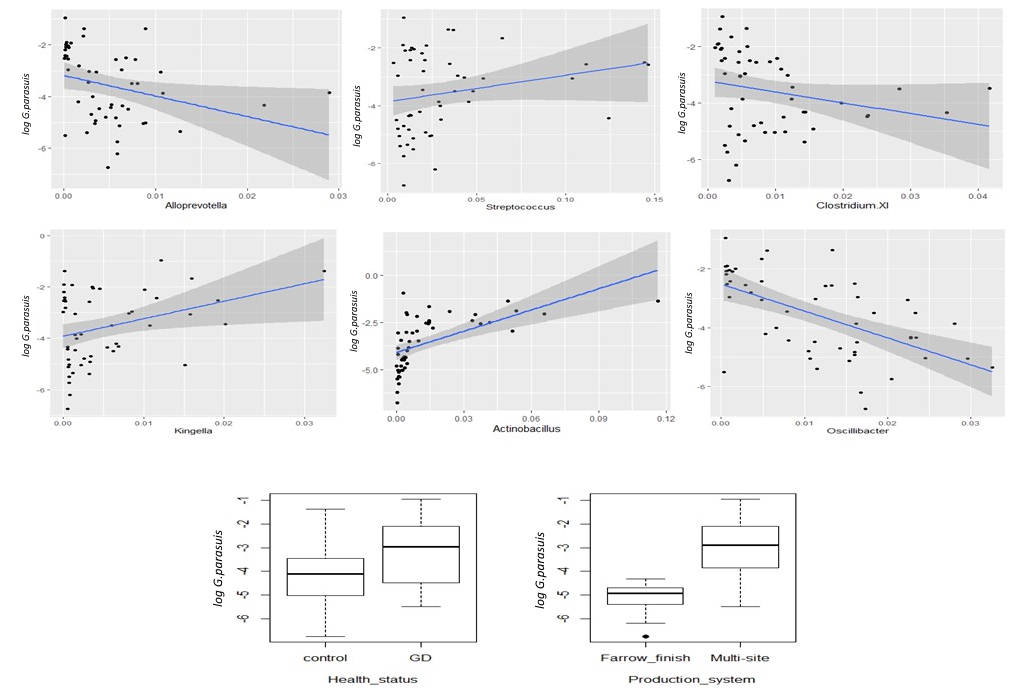
Additional file 2B Plotting the association between the relative abundance of *Glaesserella parasuis*, expressed as natural logarithm in 51 piglets and significantly variables (*P* ≤ 0.05) presented in the final model including farm management factors (production system and health status) and nasal microbiota members at genus level (*Alloprevotella*, *Streptococcus*, *Clostridium.XI*, *Oscillibacter*, *Kingella* and *Actinobacillus*)
