# Supplementary material for: Variations in association of nasal microbiota with virulent and non-virulent strains of Glaesserella (Haemophilus) parasuis in weaning piglets
Source: Vet Res. 2020 Feb 3;51:7. doi: 10.1186/s13567-020-0738-8 (PMC6996185; doi:10.1186/s13567-020-0738-8)
Supplement: Supplementary file 3 — Additional file 3. The results of univariable model analyses of virulent strain (A), non-virulent strain (B), and relative abundance of G. parasuis (C), expressed as natural logarithm at genus level taxa. A Univariable logistic regression for potential risk factors and nasal microbiota at genus level associated with the virulent strain of G. parasuis (virGp) infection (P-value ≤ 0.25) in 51 piglets in 7 Spanish farms. B Univariable logistic regression for potential risk factors and nasal microbiota at genus level associated with the non-virulent strain of G. parasuis (non-virGp) infection (P-value ≤ 0.25) in 51 piglets in 7 Spanish farms. C Univariable linear regression for potential risk factors and nasal microbiota at genus level associated with relative abundance of G. parasuis, expressed as natural logarithm, (P-value ≤ 0.25) in 51 piglets in 7 Spanish farms. [file 13567_2020_738_MOESM3_ESM.docx]

**Additional file 3** **The results of univariable model analyses of virulent strain (Additional file 3A), non-virulent strain (Additional file 3B), and relative abundance of *H. parasuis* (Additional file 3C), expressed as natural logarithm at genus level taxa.**

**Additional file 3A Univariable logistic regression for potential risk factors and nasal microbiota at genus level associated with the virulent strain of *H. parasuis* infection (*P-*value ≤ 0.25) in 51 piglets in 7 Spanish farms.**

| Genus/variable | Level | Estimates | *P* value |
| --- | --- | --- | --- |
| Health status | Control | Ref | 6.481e-05 |
|  | Glässer’s disease | 2.773 |  |
| Production system | Farrow to finish | Ref | 5.938e-06 |
|  | Multi-site | 19.11 |  |
| Corynebacterium | --- | 349.2066 | 0.001319 |
| *Rothia* | --- | 28.9653 | 0.5106 |
| *Bacteroides* | --- | -1.47746 | 0.9407 |
| *Barnesiella* | --- | 11.5629 | 0.4488 |
| *Odoribacter* | --- | 56.2288 | 0.5891 |
| *Alloprevotella* | --- | 17.0069 | 0.7477 |
| *Prevotella* | --- | -12.50874 | 0.3918 |
| *Alistipes* | --- | -0.05506 | 0.9983 |
| *Planobacterium* | --- | -0.00218 | 0.909 |
| *Streptophyta* | --- | 112.4003 | 0.3026 |
| *Staphylococcus* | --- | 16.1898 | 0.4366 |
| *Aerococcus* | --- | -54.59864 | 0.697 |
| *Lactobacillus* | --- | -3.34876 | 0.8673 |
| *Pediococcus* | --- | 0.03044 | 0.2762 |
| *Leuconostoc* | --- | 0.003312 | 0.8775 |
| *Streptococcus* | --- | 7.2003 | 0.3853 |
| *Clostridium.sensu.stricto* | --- | -138.6342 | 0.005986 |
| *Clostridium.XlVa* | --- | -14.5050 | 0.3967 |
| *Coprococcus* | --- | -206.5008 | 0.05032 |
| *Lachnospiracea_incertae_sedis* | --- | -239.7051 | 0.01129 |
| *Roseburia* | --- | -0.02444 | 0.2063 |
| *Clostridium.XI* | --- | -114.5084 | 0.008013 |
| *Clostridium.IV* | --- | -78.2183 | 0.2785 |
| *Faecalibacterium* | --- | 0.01241 | 0.5165 |
| *Flavonifractor* | --- | -0.01612 | 0.4007 |
| *Oscillibacter* | --- | -58.1450 | 0.07828 |
| *Pseudoflavonifractor* | --- | 0.02214 | 0.251 |
| *Ruminococcus* | --- | -485.9752 | 0.000186 |
| *Phascolarctobacterium* | --- | -8.4884 | 0.9559 |
| *Fusobacterium* | --- | -62.97189 | 0.2705 |
| *Bordetella* | --- | -0.02088 | 0.4554 |
| *Kingella* | --- | 45.1584 | 0.3021 |
| *Neisseria* | --- | -0.7887 | 0.9552 |
| *Arcobacter* | --- | 0.003786 | 0.8802 |
| *Succinivibrio* | --- | 0.006938 | 0.7217 |
| *Escherichia.Shigella* | --- | -693.0167 | 2.104e-05 |
| *Klebsiella* | --- | -15.1713 | 0.08251 |
| *Actinobacillus* | --- | 16.5234 | 0.2305 |
| *Pasteurella* | --- | -0.01762 | 0.3619 |
| *Acinetobacter* | --- | 7.8694 | 0.6003 |
| *Moraxella* | --- | -1.7700 | 0.3276 |
| *Pseudomonas* | --- | 77.2809 | 0.5348 |
| *Treponema* | --- | -436.8943 | 0.05454 |
| *Mycoplasma* | --- | -0.007277 | 0.7029 |

**Additional file 3B Univariable logistic regression for potential risk factors and nasal microbiota at genus level associated with the non-virulent strain of *H. parasuis* infection (*P-*value ≤ 0.25) in 51 piglets in 7 Spanish farms.**

| Genus/variable | Level | Estimates | *P* value |
| --- | --- | --- | --- |
| Health status | Control | Ref | 0.007276 |
|  | Glässer’s disease | -1.6835 |  |
| Production system | Farrow to finish | Ref | 0.01102 |
|  | Multi-site | 1.7430 |  |
| *Corynebacterium* | --- | -178.4388 | 0.0134 |
| *Rothia* | --- | -66.1213 | 0.1554 |
| *Bacteroides* | --- | -91.3217 | 0.0003216 |
| *Barnesiella* | --- | -69.3347 | 0.0005525 |
| *Odoribacter* | --- | -701.9016 | 1.06e-05 |
| *Alloprevotella* | --- | -43.2524 | 0.4234 |
| *Prevotella* | --- | 7.81439 | 0.5847 |
| *Alistipes* | --- | -169.9292 | 1.068e-05 |
| *Planobacterium* | --- | 6.83566 | 0.00434 |
| *Streptophyta* | --- | -125.2814 | 0.2541 |
| *Staphylococcus* | --- | 6.12322 | 0.762 |
| *Aerococcus* | --- | 142.17980 | 0.3386 |
| *Lactobacillus* | --- | 12.43510 | 0.540 |
| *Pediococcus* | --- | 8.38158 | 0.1623 |
| *Leuconostoc* | --- | 1.646 | 0.1424 |
| *Streptococcus* | --- | -6.4791 | 0.4329 |
| *Clostridium.sensu.stricto* | --- | -3.2779 | 0.936 |
| *Clostridium.XlVa* | --- | -56.4820 | 0.003301 |
| *Coprococcus* | --- | -12.2353 | 0.894 |
| *Lachnospiracea_incertae_sedis* | --- | -33.8224 | 0.6869 |
| *Roseburia* | --- | 5.1335 | 0.9544 |
| *Clostridium.XI* | --- | 5.41389 | 0.8688 |
| *Clostridium.IV* | --- | -309.0804 | 0.0002872 |
| *Faecalibacterium* | --- | -6.9365 | 0.8939 |
| *Flavonifractor* | --- | -0.8929 | 0.02747 |
| *Oscillibacter* | --- | -97.4115 | 0.00483 |
| *Pseudoflavonifractor* | --- | 2.95169 | 0.6335 |
| *Ruminococcus* | --- | 35.645508 | 0.6874 |
| *Phascolarctobacterium* | --- | 481.2826 | 0.03551 |
| *Fusobacterium* | --- | 143.0987 | 0.05816 |
| *Bordetella* | --- | 18.4712 | 0.1799 |
| *Kingella* | --- | -19.6502 | 0.6454 |
| *Neisseria* | --- | 24.9392 | 0.1283 |
| *Arcobacter* | --- | 66.19146 | 0.1281 |
| *Succinivibrio* | --- | 0.1608 | 0.3519 |
| *Escherichia.Shigella* | --- | 19.10798 | 0.2981 |
| *Klebsiella* | --- | -4.1031 | 0.2978 |
| *Actinobacillus* | --- | 2.20731 | 0.8654 |
| *Pasteurella* | --- | -16.7797 | 0.4584 |
| *Acinetobacter* | --- | 57.0601 | 0.02236 |
| *Moraxella* | --- | 0.12580 | 0.9437 |
| *Pseudomonas* | --- | -22.8618 | 0.8475 |
| *Treponema* | --- | 381.2991 | 0.07723 |
| *Mycoplasma* | --- | -11.7451 | 0.6335 |

**Additional file 3C Univariable linear regression for potential risk factors and nasal microbiota at genus level associated with relative abundance of *H. parasuis*, expressed as natural logarithm, (*P-*value ≤ 0.25) in 51 piglets in 7 Spanish farms.**

| Genus | Level | Estimate | St. Error | *P* value |
| --- | --- | --- | --- | --- |
| Health status | Control | Ref | Ref | 0.03325 |
|  | Glässer’s disease | 0.8702 | 0.3972 |  |
| Production system | Farrow to finish | Ref | Ref | 1.993e-07 |
|  | Multi-site | 2.0880 | 0.3454 |  |
| *Corynebacterium* | --- | -3.6569 | 27.1644 | 0.893 |
| *Rothia* | --- | -13.4209 | 30.8749 | 0.666 |
| *Bacteroides* | --- | -48.4193 | 12.2464 | 0.0002472 |
| *Barnesiella* | --- | -32.3000 | 9.7020 | 0.00166 |
| *Odoribacter* | --- | -223.9929 | 66.3197 | 0.001441 |
| *Alloprevotella* | --- | -79.3884 | 35.6777 | 0.03071 |
| *Prevotella* | --- | -7.7589 | 9.8910 | 0.437 |
| *Alistipes* | --- | -63.8886 | 15.4933 | 0.000144 |
| *Planobacterium* | --- | 24.7805 | 10.5973 | 0.0235 |
| *Streptophyta* | --- | -45.8553 | 74.8481 | 0.543 |
| *Staphylococcus* | --- | 1.8778 | 14.0971 | 0.8946 |
| *Aerococcus* | --- | -88.1784 | 97.0794 | 0.368 |
| *Lactobacillus* | --- | -17.1054 | 13.9548 | 0.2261 |
| *Pediococcus* | --- | -63.3563 | 81.1719 | 0.4388 |
| *Leuconostoc* | --- | 16.0788 | 54.0957 | 0.768 |
| *Streptococcus* | --- | 9.1484 | 5.6411 | 0.1113 |
| *Clostridium.sensu.stricto* | --- | -21.0588 | 28.7898 | 0.468 |
| *Clostridium.XlVa* | --- | -38.4424 | 10.6050 | 0.0006866 |
| *Coprococcus* | --- | -44.3138 | 64.8611 | 0.4977 |
| *Lachnospiracea_incertae_sedis* | --- | -105.0744 | 57.5431 | 0.07394 |
| *Roseburia* | --- | -67.1106 | 62.2268 | 0.2861 |
| *Clostridium.XI* | --- | -38.2773 | 22.5119 | 0.09541 |
| *Clostridium.IV* | --- | -176.2099 | 43.2913 | 0.0001706 |
| *Faecalibacterium* | --- | 40.6291 | 75.5526 | 0.5932 |
| *Flavonifractor* | --- | -169.6025 | 39.3974 | 7.979e-05 |
| *Oscillibacter* | --- | -91.2708 | 18.3915 | 8.792e-06 |
| *Pseudoflavonifractor* | --- | -171.0608 | 43.0137 | 0.0002297 |
| *Ruminococcus* | --- | -70.1734 | 61.5631 | 0.2599 |
| *Phascolarctobacterium* | --- | 4.6001 | 108.6165 | 0.9664 |
| *Fusobacterium* | --- | 5.4652 | 34.7393 | 0.8756 |
| *Bordetella* | --- | 41.7197 | 30.5706 | 0.1786 |
| *Kingella* | --- | 68.2099 | 28.5844 | 0.02093 |
| *Neisseria* | --- | 2.1411 | 9.9466 | 0.8305 |
| *Arcobacter* | --- | 55.2204 | 55.4257 | 0.324 |
| *Succinivibrio* | --- | -36.6303 | 104.4141 | 0.7272 |
| *Escherichia.Shigella* | --- | -8.7174 | 10.1857 | 0.3962 |
| *Klebsiella* | --- | -3.1701 | 2.3401 | 0.1817 |
| *Actinobacillus* | --- | 37.4425 | 7.4768 | 7.532e-06 |
| *Pasteurella* | --- | -5.3938 | 8.5015 | 0.5287 |
| *Acinetobacter* | --- | 20.6807 | 10.1609 | 0.04725 |
| *Moraxella* | --- | -0.9524 | 1.2544 | 0.4514 |
| *Pseudomonas* | --- | 21.8795 | 84.1640 | 0.796 |
| *Treponema* | --- | -84.8272 | 105.4021 | 0.4248 |
| *Mycoplasma* | --- | -6.0889 | 3.4299 | 0.08207 |
